# Supplementary material for: DMRforPairs: identifying Differentially Methylated Regions between unique samples using array based methylation profiles
Source: BMC Bioinformatics. 2014 May 15;15:141. doi: 10.1186/1471-2105-15-141 (PMC4046028; doi:10.1186/1471-2105-15-141)
Supplement: Additional file 2 — DMRforPairs output for the comparison of A431-MCF7 and NA17018-NA17105. Please start from the HTML files in each folder. Available via the BMC Bioinformatics website. [file 1471-2105-15-141-S2.zip › 1394847754114233_MOESM2_ESM/A431_MCF7/figures/37493.pdf]

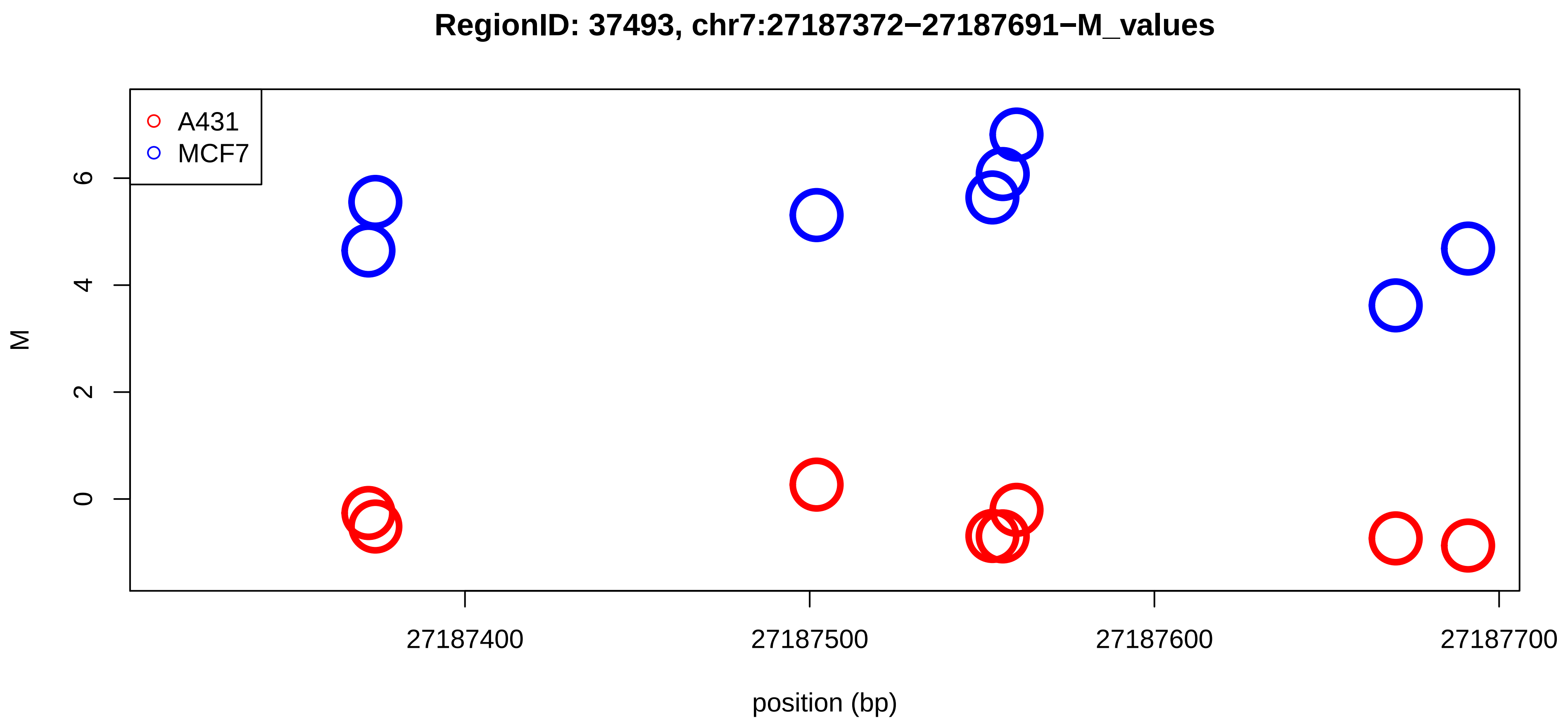

RegionID: 37493, chr7:27187372−27187691−M_values
A431
MCF7
M
0
2
4
6
27187400
27187500
27187600
27187700
position (bp)

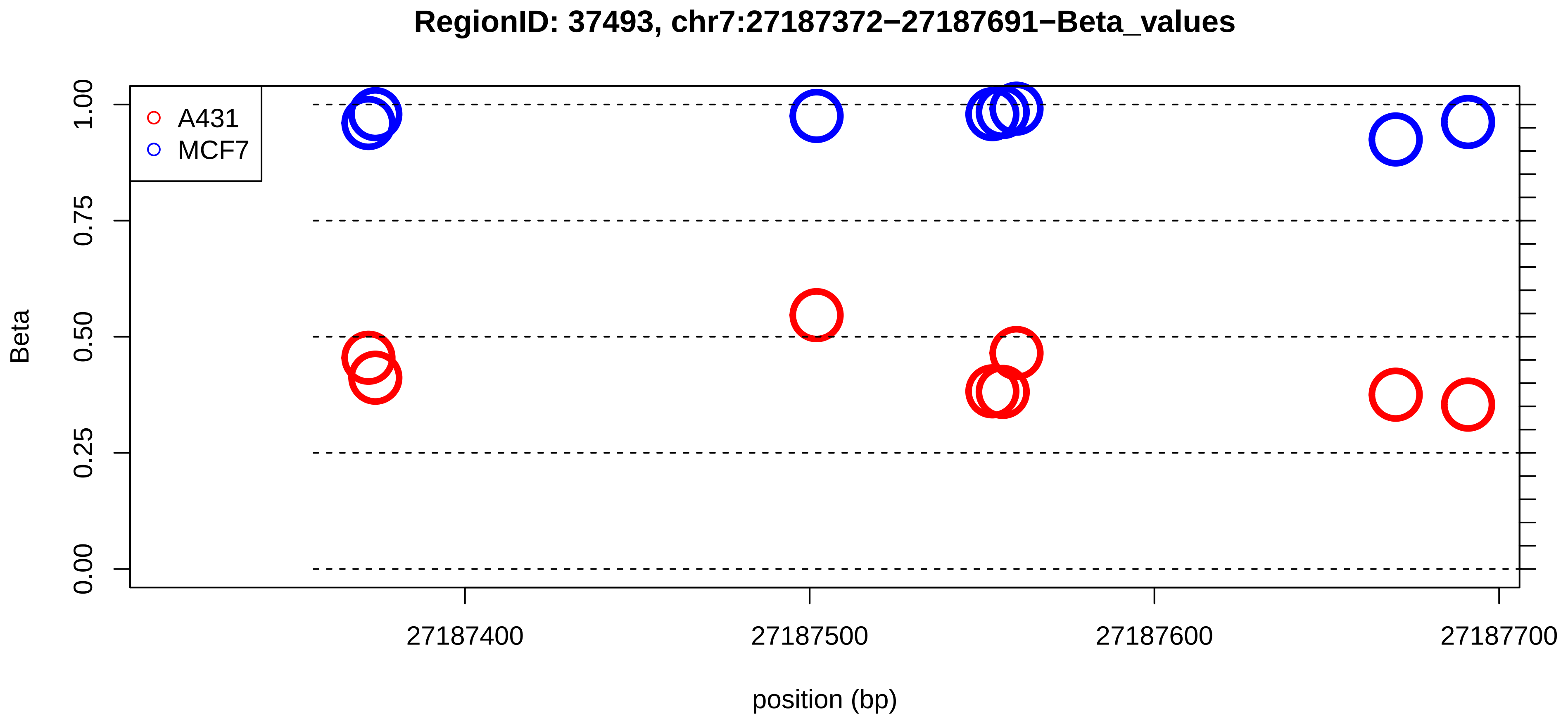

RegionID: 37493, chr7:27187372−27187691−Beta_values
A431
MCF7
Beta
0.00
0.25
0.50
0.75
1.00
27187400
27187500
27187600
27187700
position (bp)

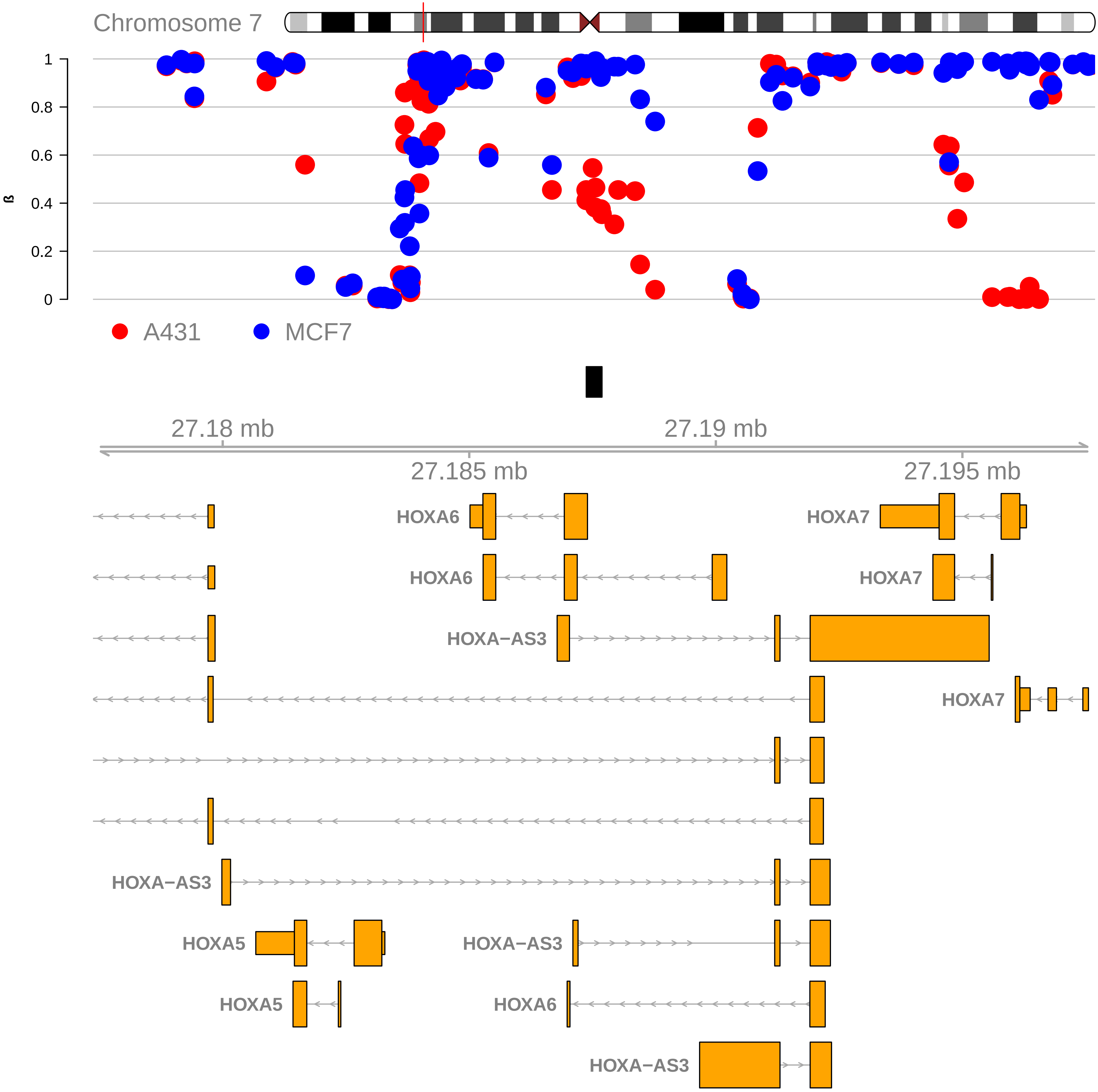

Chromosome 7
ß
1
0.8
0.6
0.4
0.2
0
A431
MCF7
27.18 mb
27.19 mb
27.185 mb
27.195 mb
HOXA6
HOXA7
HOXA6
HOXA7
HOXA−AS3
HOXA7
HOXA−AS3
HOXA5
HOXA−AS3
HOXA5
HOXA6
HOXA−AS3
